# Colovesical Fistula: An Uncommon Cause of Hematuria and Rectal Bleeding

**DOI:** 10.1155/2022/1419250

**Published:** 2022-11-02

**Authors:** Jeffrey K. Than, Greg S. Cohen

**Affiliations:** ^1^Department of Medicine, McGaw Medical Center of Northwestern University, Chicago, IL, USA; ^2^Division of Gastroenterology and Hepatology, Department of Medicine, Northwestern University, Chicago, Illinois, USA

## Abstract

Colovesical fistula is an infrequent complication of diverticular disease that presents with pneumaturia, fecaluria, dysuria and, rarely, hematuria or hematochezia. Here we present a case of concurrent hematuria and rectal bleeding arising from a diverticular bleed traversing a previously undiagnosed colovesical fistula. Other causes of colovesical fistula include Crohn's disease, radiation, and malignancy, though it is most commonly caused by complicated diverticulitis as in this case. Computed tomography (CT) imaging, cystoscopy, and gastrograffin enema have been described as high-yield diagnostic tests. Interestingly, colonoscopy is only successful in diagnosing colovesical fistula in approximately 55% of cases. Management often requires surgical intervention, as in this case, given limited success with conservative management. Colovesical fistula should be considered in patients presenting with fecaluria, pneumaturia, and dysuria as well as in cases of hematuria.

## 1. Introduction

Colovesical fistula is an uncommon complication of complicated diverticular disease with an estimated prevalence between 2% and 23% [1]. Patients with a colovesical fistula commonly present with pneumaturia, fecaluria, recurrent urinary tract infection (UTI), and symptoms of urinary frequency, though they can rarely present with hematuria. Other etiologies of colovesical fistula include fistulizing Crohn's disease, malignancy, or history of radiation [2]. Here we describe an individual presenting with both hematuria and hematochezia who was found to have a colovesical fistula in the setting of complex diverticular disease.

## 2. Case Report

A 66-year-old man presented to the emergency department with acute-onset gross hematuria and rectal bleeding. His medical history was notable for sigmoid and descending colon diverticulosis seen on colonoscopy in 2019 and benign prostatic hyperplasia (BPH), for which he underwent greenlight laser transurethral resection of the prostate 5 months prior. His postoperative course was notable for urinary tract infection, acute urinary retention, and persistent pneumaturia.

**Figure 1:**
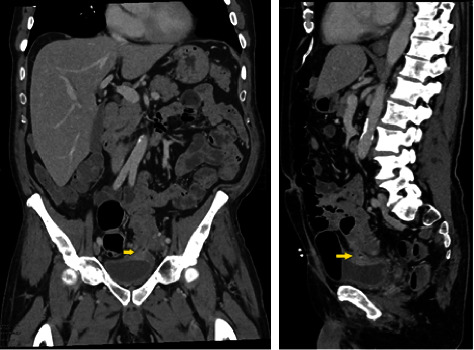
Sagittal and coronal CT imaging of colovesical fistula extending from the sigmoid colon to the left mid bladder.

Computed tomography (CT) of the abdomen and pelvis revealed sigmoid and bladder wall thickening, intravesicular gas, and a previously undiagnosed colovesical fistula (shown in Figure 1). The CT was also notable for abnormal prostatic enhancement, iliac lymphadenopathy, and diffuse osseous metastatic disease suggestive of metastatic prostate adenocarcinoma which was later confirmed on prostate biopsy.

**Figure 2:**
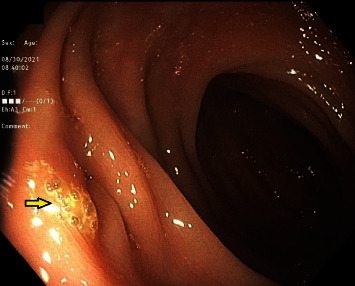
Endoscopic image of an area of indurated tissue 20 cm from the anal verge concerning the fistula.

Colonoscopy showed old blood, sigmoid diverticulosis, and an area of indurated tissue 20 centimeters from the anal verge concerning for the fistula (shown in Figure 2). His presentation was consistent with a diverticular bleed that traversed the fistula, causing both hematuria and rectal bleeding. The etiology of the fistula was likely a prior episode of diverticulitis given his sigmoid diverticulosis; the location of the fistula was far from the site of any recent urologic instrumentation. The urine culture from admission grew *Escherichia coli*, for which he was treated with cephalexin. After discharge, he underwent robotic sigmoid colectomy with colorectal anastomosis and colovesical fistula takedown.

This case highlights a unique presentation of complicated diverticular disease with concurrent hematuria and rectal bleeding caused by diverticular bleeding traversing a colovesical fistula. As in this case, management of diverticular disease complicated by fistulas relies on multidisciplinary discussion including colorectal surgery and urology given bladder involvement. While colovesical fistula rarely presents with hematuria, it should be considered, particularly in patients with known diverticulosis.

## 3. Discussion

In this patient, his colovesical fistula most likely stemmed from a prior episode of diverticulitis which is consistent with the sigmoid diverticula seen on his colonoscopy. While diverticular disease accounts for approximately two-thirds of cases of colovesical fistula, other etiologies include malignancy including bladder and colon cancer, radiation, and fistulizing Crohn's disease [2]. These patients most commonly presented with pneumaturia, urinary frequency, dysuria, fecaluria, or with fecaluria and terminal pneumaturia considered to be pathognomonic [3]. Interestingly, these patients are rarely found to have leukocytosis or fever [1]. In a retrospective chart review of 39 patients with colovesical fistula, two patients (5%) presented with rectal bleeding, and only one patient (2%) presented with hematuria [2]. Presentation with concurrent hematuria and rectal bleeding has not been described in the existing literature.

Complicated diverticulitis refers to diverticular disease associated with obstruction, abscess, perforation, fistula, or stricture [4]. These patients typically present with left lower quadrant pain, changes in bowel movements, and low-grade fever. Uncomplicated diverticulitis can often be managed conservatively with antibiotics and bowel rest. However, recurrent uncomplicated disease or complicated diverticulitis often requires surgical consultation.

Diverticulitis complicated by fistula remains a difficult entity to manage. Approximately 2/3 of diverticular fistulas are colovesical and these most commonly affect the sigmoid colon, as in the patient described above [5]. The pathogenesis includes rupture of the diverticulum or erosion of a diverticular abscess into the bladder [1]. Men are twice as likely to have a colovesical fistula than women due to the anatomical barrier of the uterus [5]. Other manifestations of diverticulitis complicated by fistula include enterocolonic fistulas which present primarily with diarrhea, and colovaginal fistulas which present with passing stool through the vagina [4].

Diagnosis of colovesical fistula in this case was made with CT imaging with contrast, though demonstrating the fistula radiographically can be challenging. The literature describes other diagnostic modalities that can be considered. In a study of 39 patients with a colovesical fistula, the diagnosis was made with CT imaging in 80% of cases. Other high-yield diagnostic modalities include cystograms, gastrograffin enema, and cystoscopy. Interestingly, the literature estimates the rate of successfully diagnosing a colovesical fistula via colonoscopy at 0% to 55%. Of note, oral charcoal administration and visualization of charcoal per urethra diagnosed colovesical fistula in all five cases when it was used [2]. The literature recommends surgical resection given the low likelihood of disease resolution with conservative management [4]. A recent meta-analysis demonstrated a shorter hospital length of stay with similar operating time, postoperative complication rates, and mortality rates in laparoscopic compared to open repair [6].

This case highlights an unusual presentation of colovesical fistula in a patient with sigmoid diverticulosis. Colovesical fistula should be considered in patients presenting with pneumaturia, fecaluria, or recurrent urinary tract infection.

## Data Availability

No data were used to support this study.
